# Supplementary material for: PCAF‐mediated acetylation of ISX recruits BRD4 to promote epithelial‐mesenchymal transition
Source: EMBO Rep. 2020 Jan 7;21(2):e48795. doi: 10.15252/embr.201948795 (PMC7001155; doi:10.15252/embr.201948795)
Supplement: Supplementary file 1 — Expanded View Figures PDF [file EMBR-21-e48795-s001.pdf]

Expanded View Figures

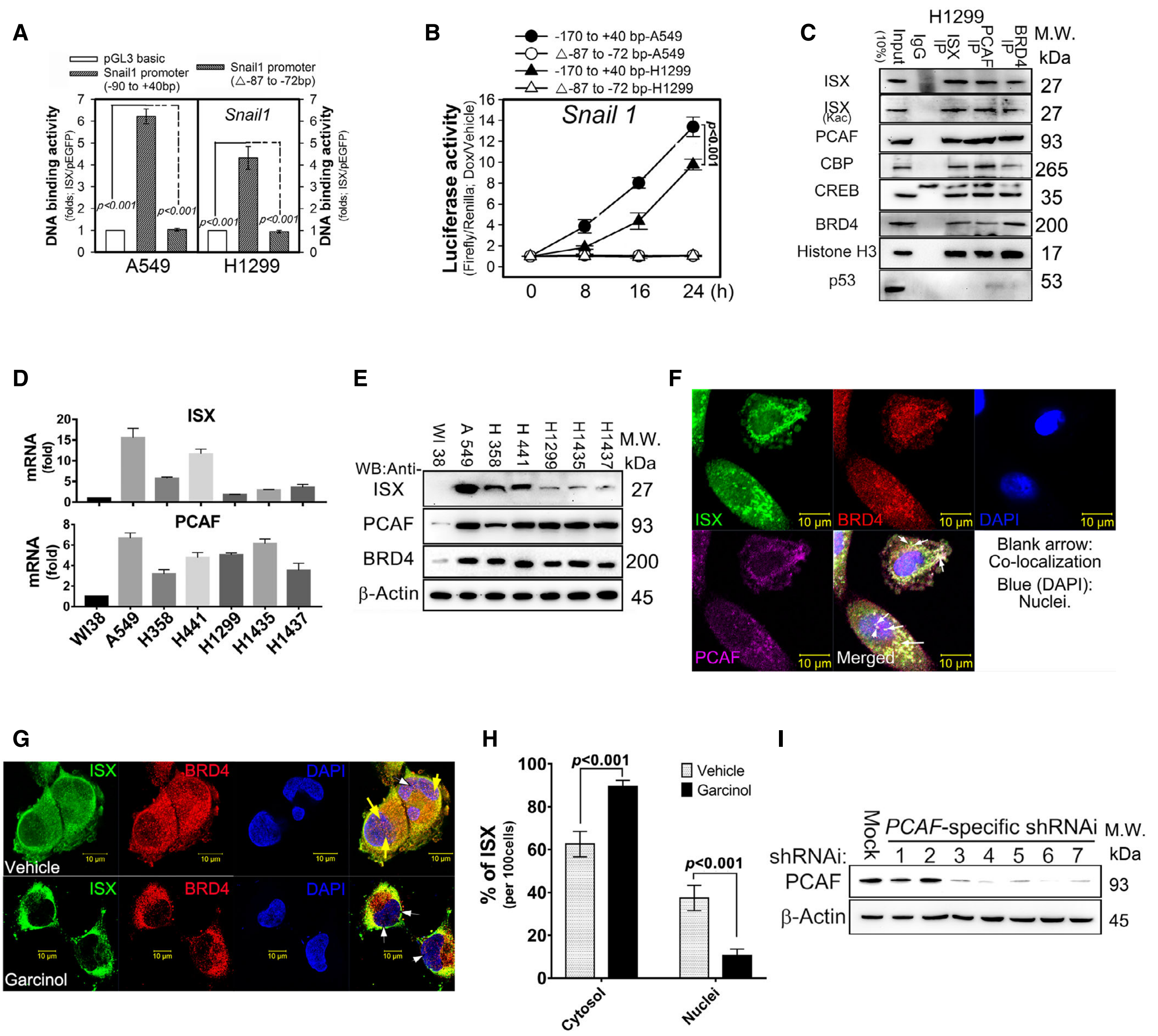

Figure EV1.

© 2020 The Authors

Figure EV1. PCAF modulated EMT characteristics induced by the ISX-BRD4 complex.

A ChIP analysis of ISX binding to the promoters of Snail1 in A549 and H1299 cells. Data are presented as mean ± SD in bar graph ($P < 0.001$, Student's *t*-test) of three independent experiments, each performed in triplicate.

B ISX transactivation activity analyzed by luciferase activity driven by the Snail1 promoter. Data are presented as mean ± SD in graph ($P < 0.001$, Student's *t*-test) of three independent experiments, each performed in triplicate.

C ISX-associated proteins (PCAF, BRD4, CBP, CREB, and histone H3) determined by Western blotting in immunoprecipitation of H1299 cells.

D, E Endogenous mRNA and protein expression of ISX, PCAF, and BRD4 was determined in various lung cancer cell lines. Data are presented as mean ± SD in graph ($P < 0.001$, Student's *t*-test) of three independent experiments, each performed in triplicate.

F Confocal immunofluorescence detection of ISX (green), BRD4 (red), and PCAF (pink) in A549 cells. Cell nuclei were visualized by DAPI (blue). Blank arrow, co-localization.

G Confocal immunofluorescence detection of ISX (green) and BRD4 (red) localization in A549 cells treated with Garcinol. Cell nuclei were visualized by DAPI (blue). Yellow arrows indicate co-localization in nuclei, and white arrows indicate co-localization in the cytosol.

H A quantification of ISX in cytosol and nuclei. Data are presented as mean ± SD in bar graph ($P < 0.001$, Student's *t*-test) of three independent experiments, each performed in triplicate.

I PCAF expression in A549 cells was knocked down with 7 sequence-specific shRNAi constructs.

Data information: Each experiment was repeated at least three times.
Source data are available online for this figure.

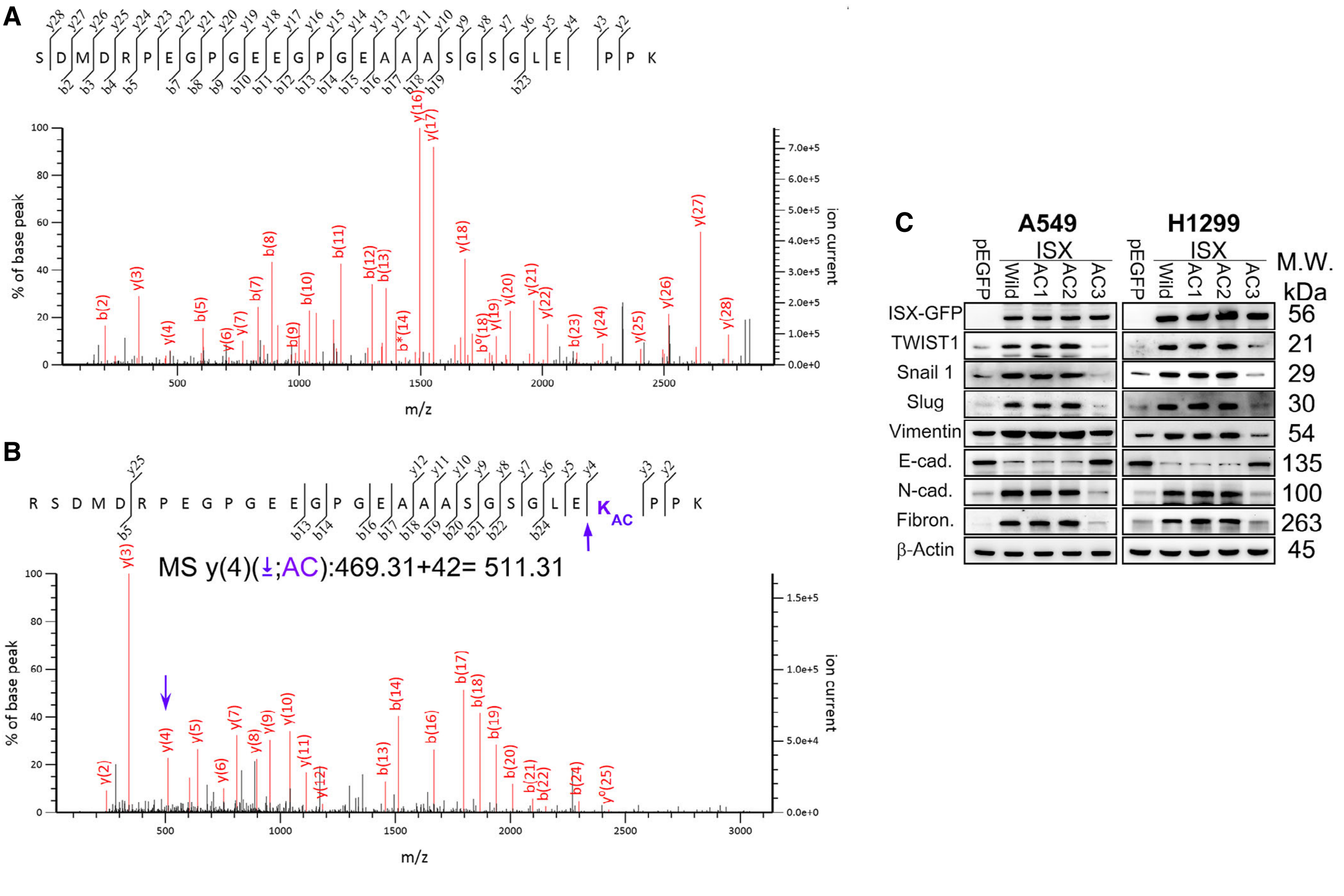

Figure EV2. Sequencing analysis of acetylated recombinant and trypsin-digested wild-type ISX using liquid chromatography-mass spectrometry.

A, B PCAF acetylation of ISX was detected at lysine residue 69 by liquid chromatography–tandem mass spectrometry (LC-tandem MS-MS). The peptide NH_2-SDMDRPEGPGEEGPGEAAASGSGLEKPPK-COOH of ISX (amino acids 44–72 a.a.) was identified with acetylated lysine at position 69 (y4, arrow).

C Expression levels of EMT markers were detected in A549 and H1299 cells transfected with wild-type and mutants of ISX cDNA by Western blotting.

Source data are available online for this figure.

 © 2020 The Authors

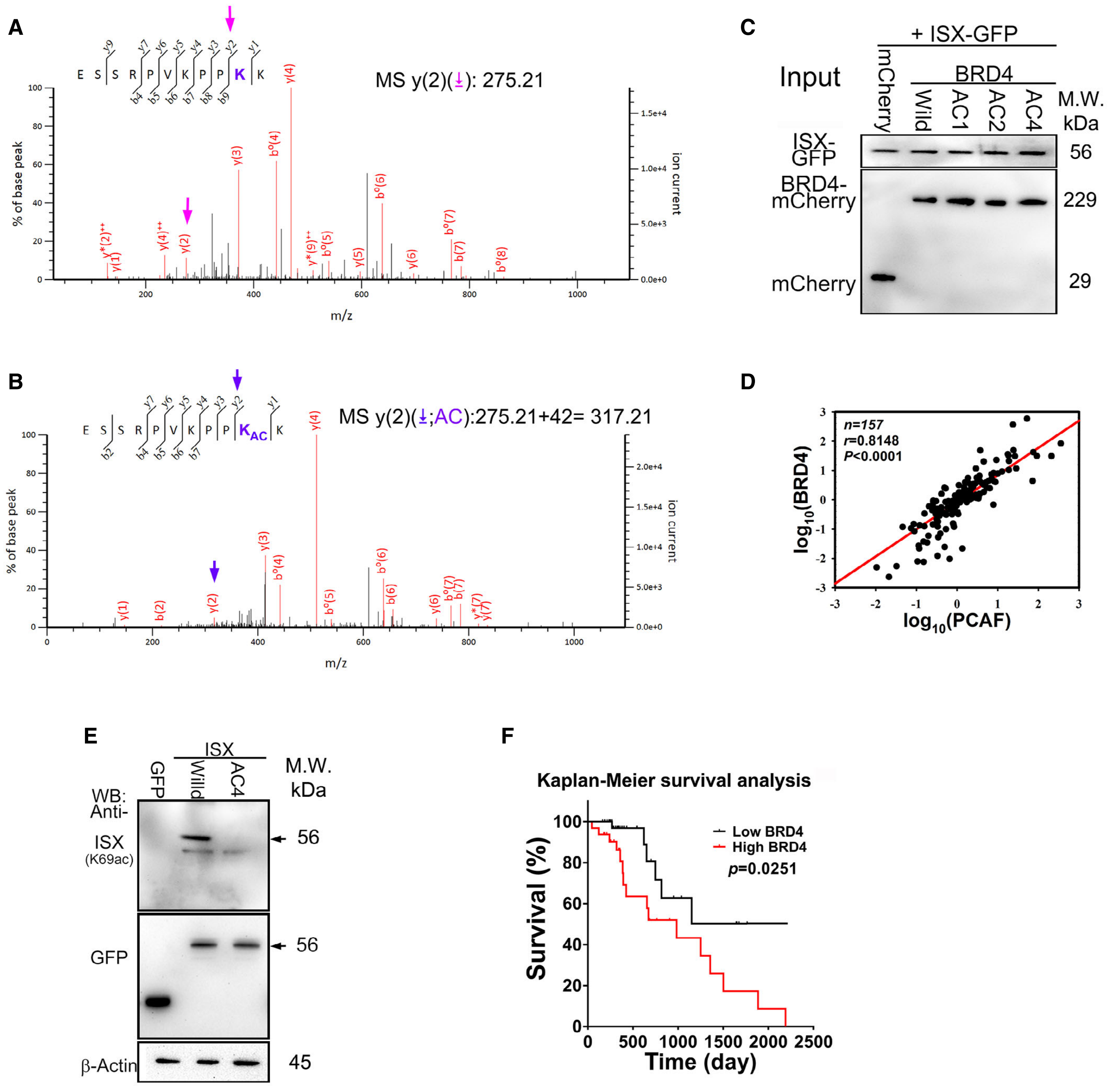

Figure EV3. Sequencing analysis of acetylated recombinant and trypsin-digested wild-type BRD4 using liquid chromatography-mass spectrometry.

A, B PCAF acetylation of BRD4 is detected at lysine residue 332 by liquid chromatography–tandem mass spectrometry (LC-tandem MS-MS). The peptide NH_2-ESSRPVKPPKK-COOH of BRD4 (amino acids 323–333 a.a.) was identified with acetylation lysine acetylated at position 332 (y(2), arrow).

C Ectopic expression of ISX-GFP and wild-type and mutants of BRD4 was detected by Western blotting in A549 cells.

D Correlation between BRD4 and PCAF mRNA levels detected by real-time PCR in 157 lung cancer samples.

E The antibody generated by acetylated ISX-K69 peptide detects GFP-tagged wild-type ISX, but not GFP-tagged ISX-K69 mutant.

F The Kaplan–Meier survival curve was used to analyze survival correlation between patients with NSCLC (*n* = 157) and BRD4. On the basis of the cut-off values of fold differences, the study population was dichotomized into "high" and "low" expression groups. *P*-values were calculated by log-rank (Mantel–Cox) test comparing the two Kaplan–Meier curves.

Source data are available online for this figure.

© 2020 The Authors

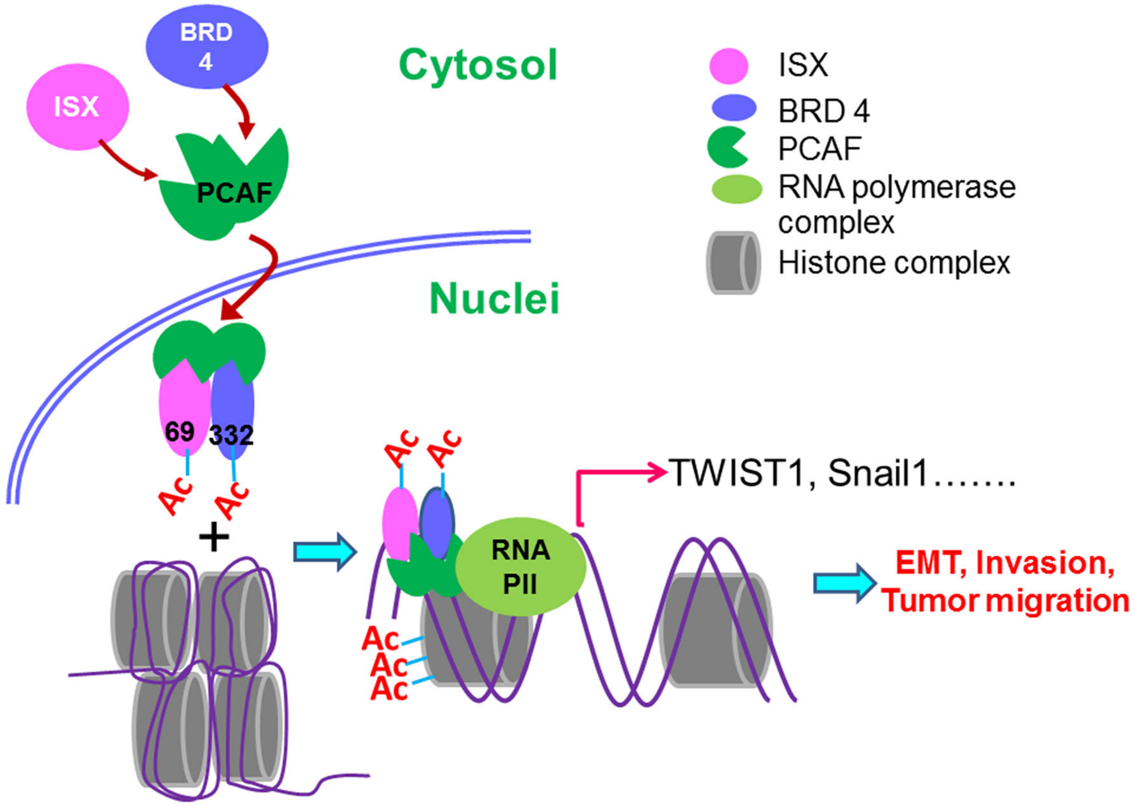

Figure EV4. Model for the ISX–BRD4–PCAF axis in lung cancer metastasis.

 © 2020 The Authors
