# Supplementary figures and images for: PCAF‐mediated acetylation of ISX recruits BRD4 to promote epithelial‐mesenchymal transition
Source: EMBO Rep. 2020 Jan 7;21(2):e48795. doi: 10.15252/embr.201948795 (PMC7001155; doi:10.15252/embr.201948795)

H1299

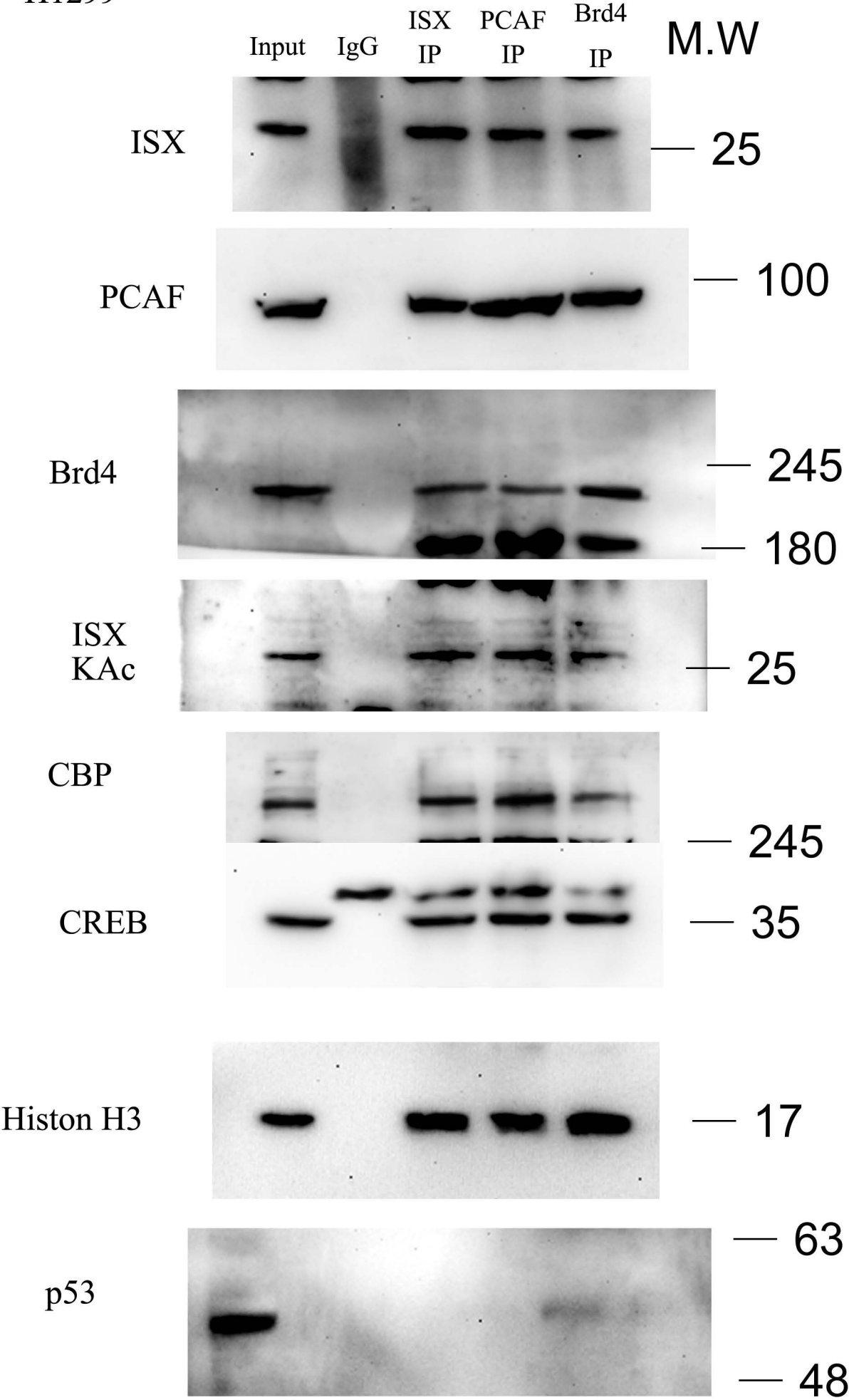

Figure EV1c

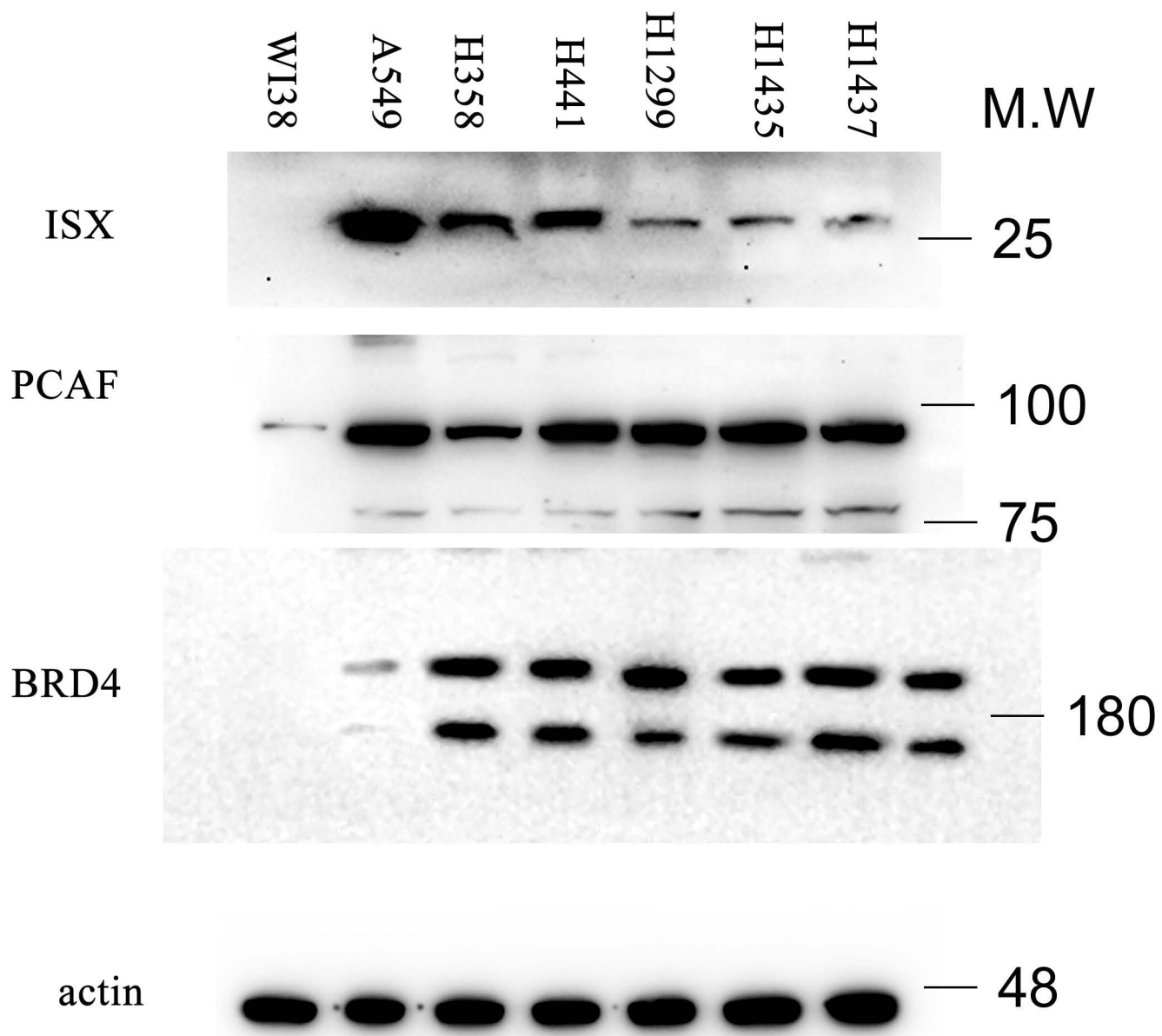

Figure EV1e

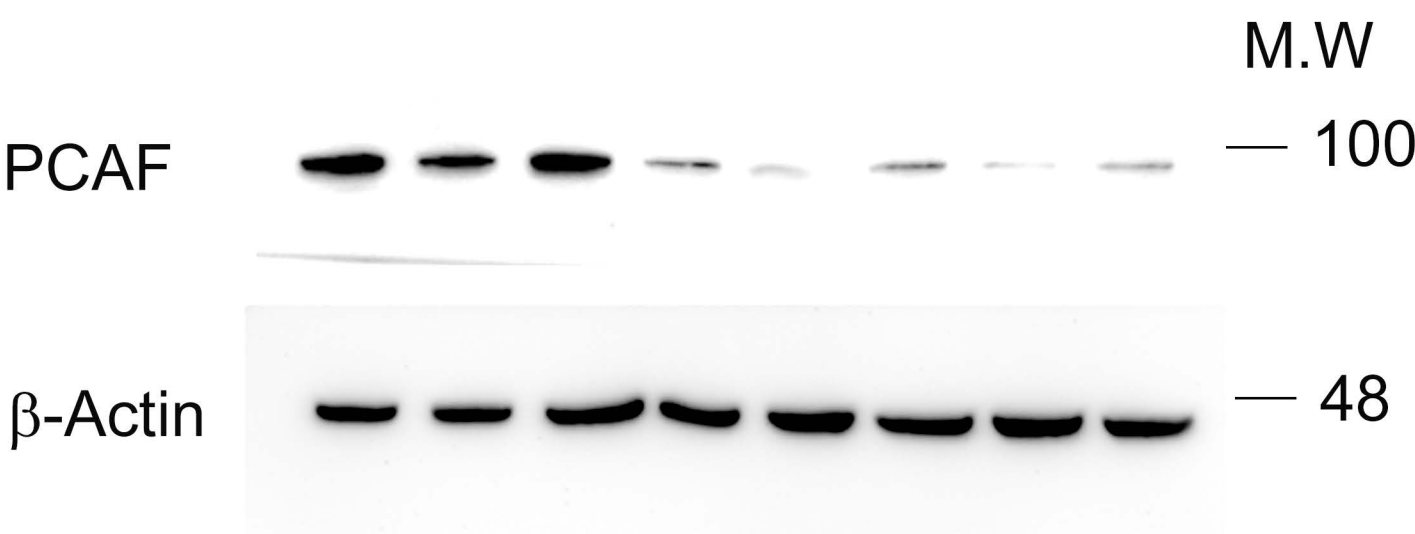

Figure EV1i

Supplement: Supplementary file 2 — Source Data for Expanded View [file EMBR-21-e48795-s009.zip › Source_Data_for_EV_Figures/Source_Data_Figure_EV1.pdf]

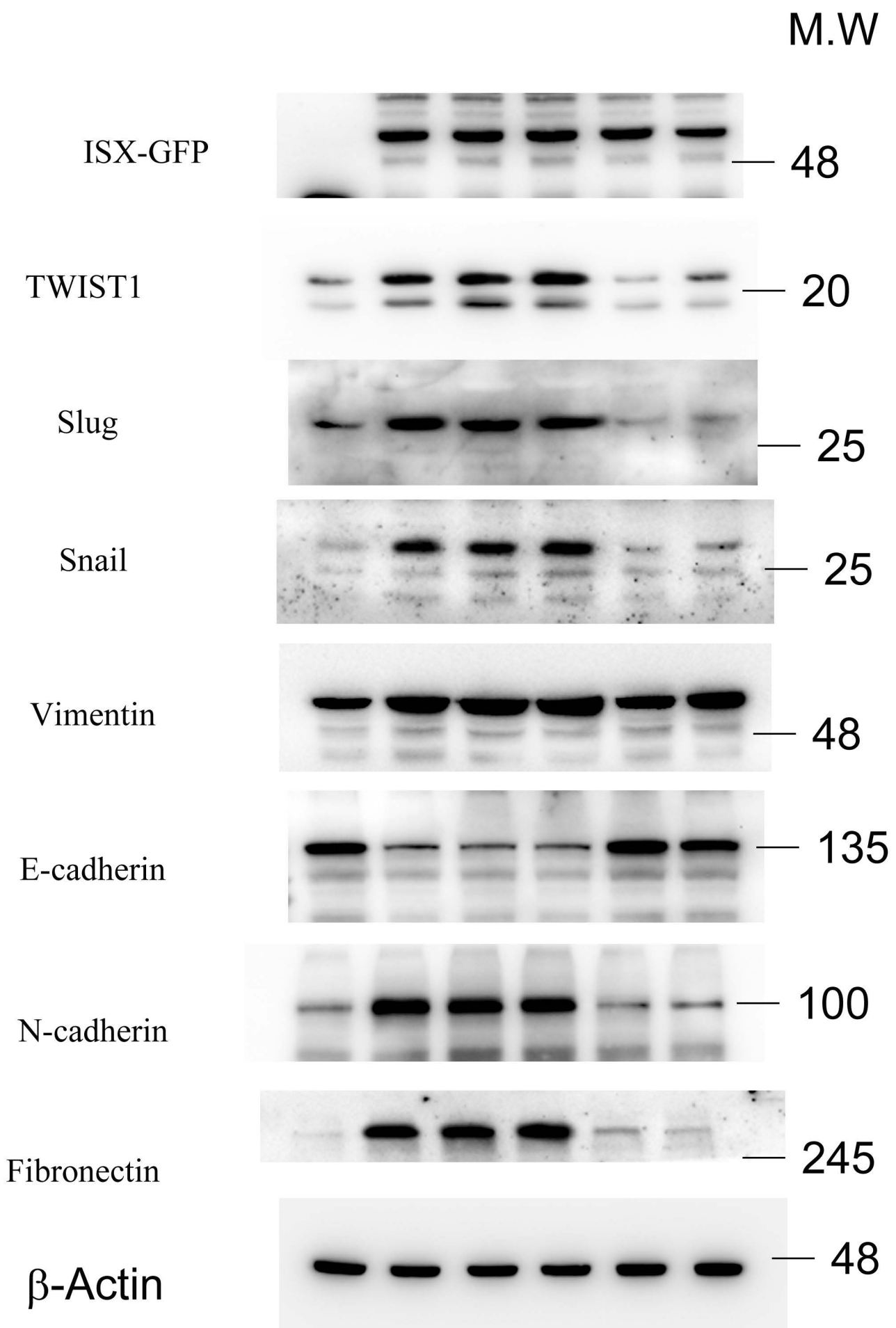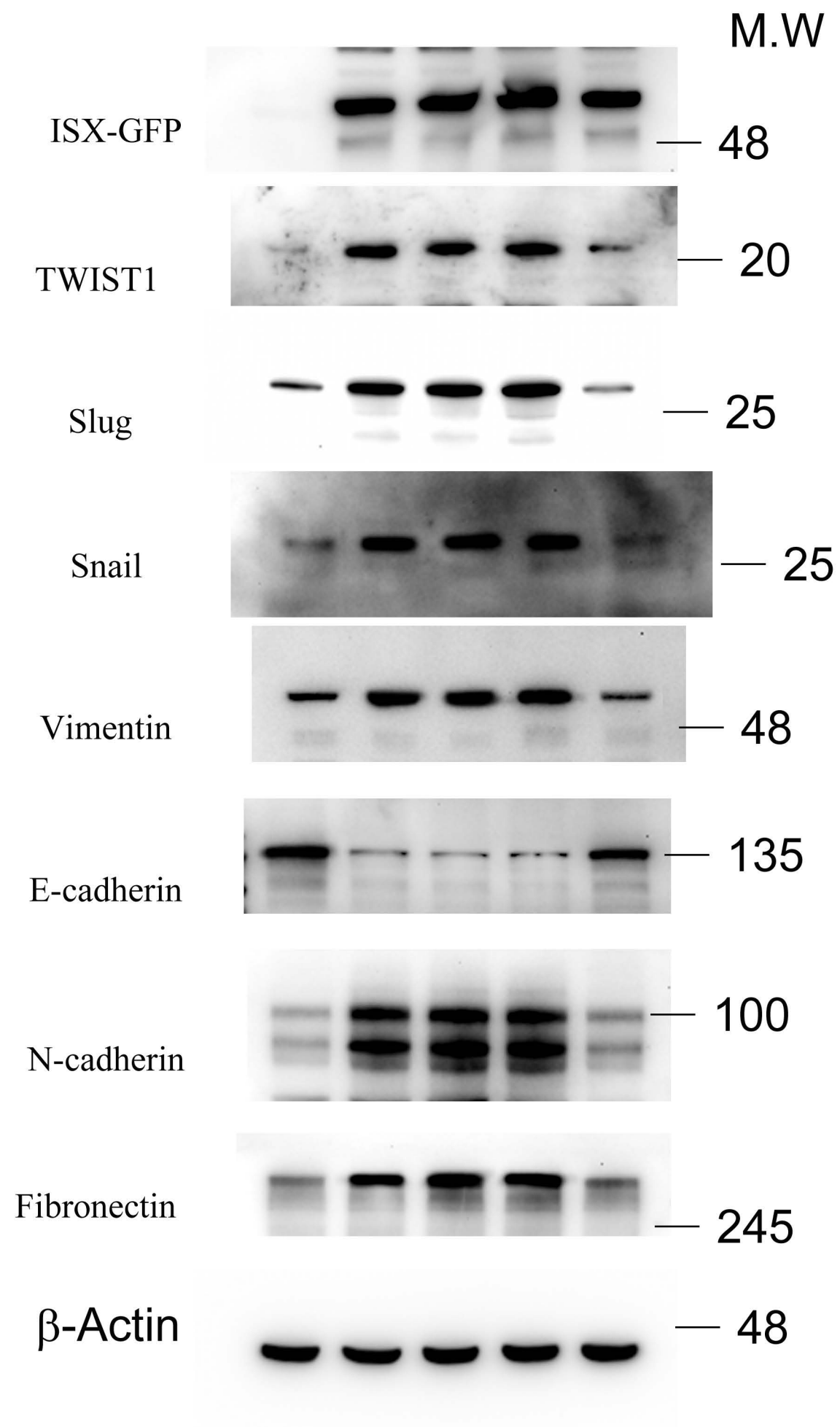

Figure EV2c

Supplement: Supplementary file 2 — Source Data for Expanded View [file EMBR-21-e48795-s009.zip › Source_Data_for_EV_Figures/Source_Data_Figure_EV2.pdf]

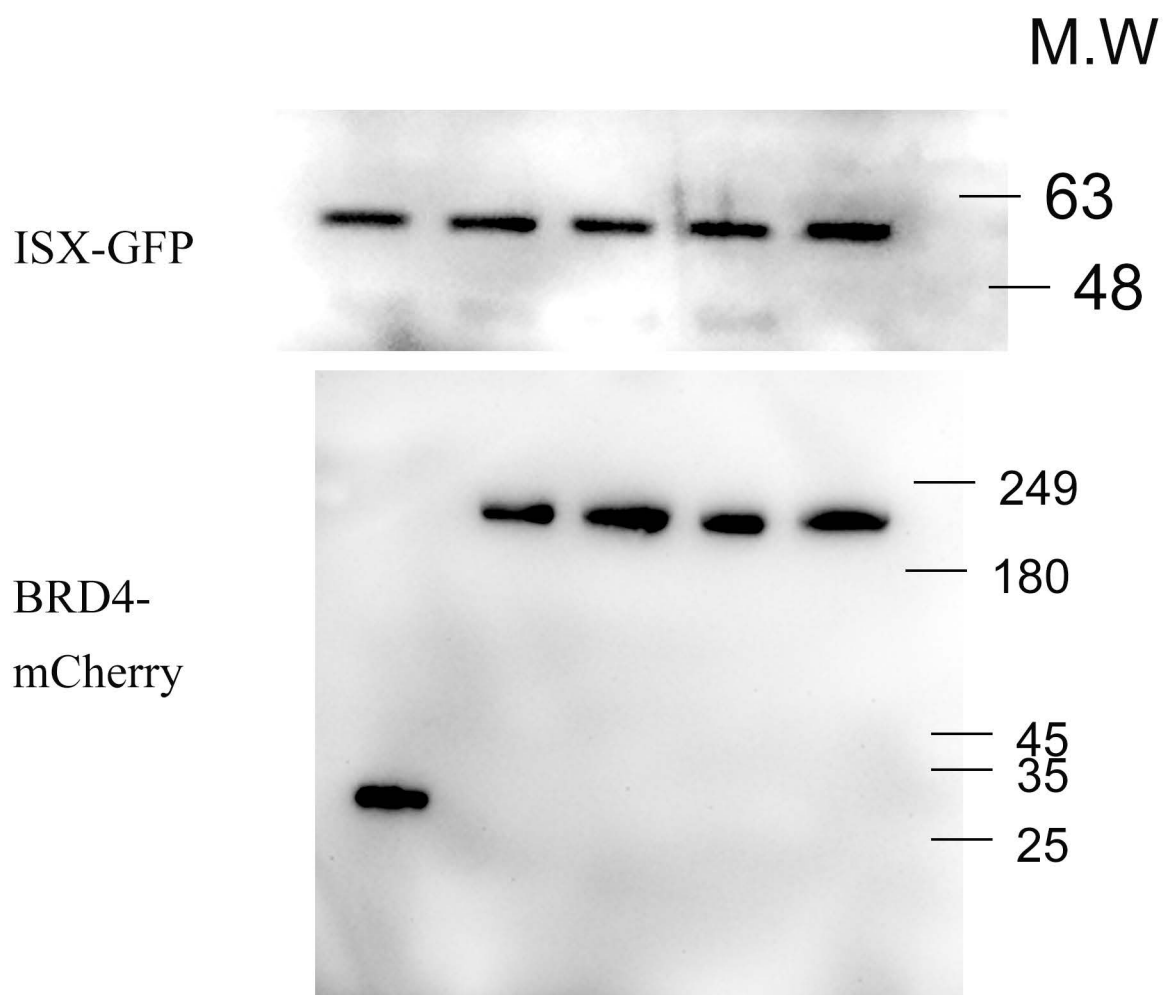

Figure EV3c

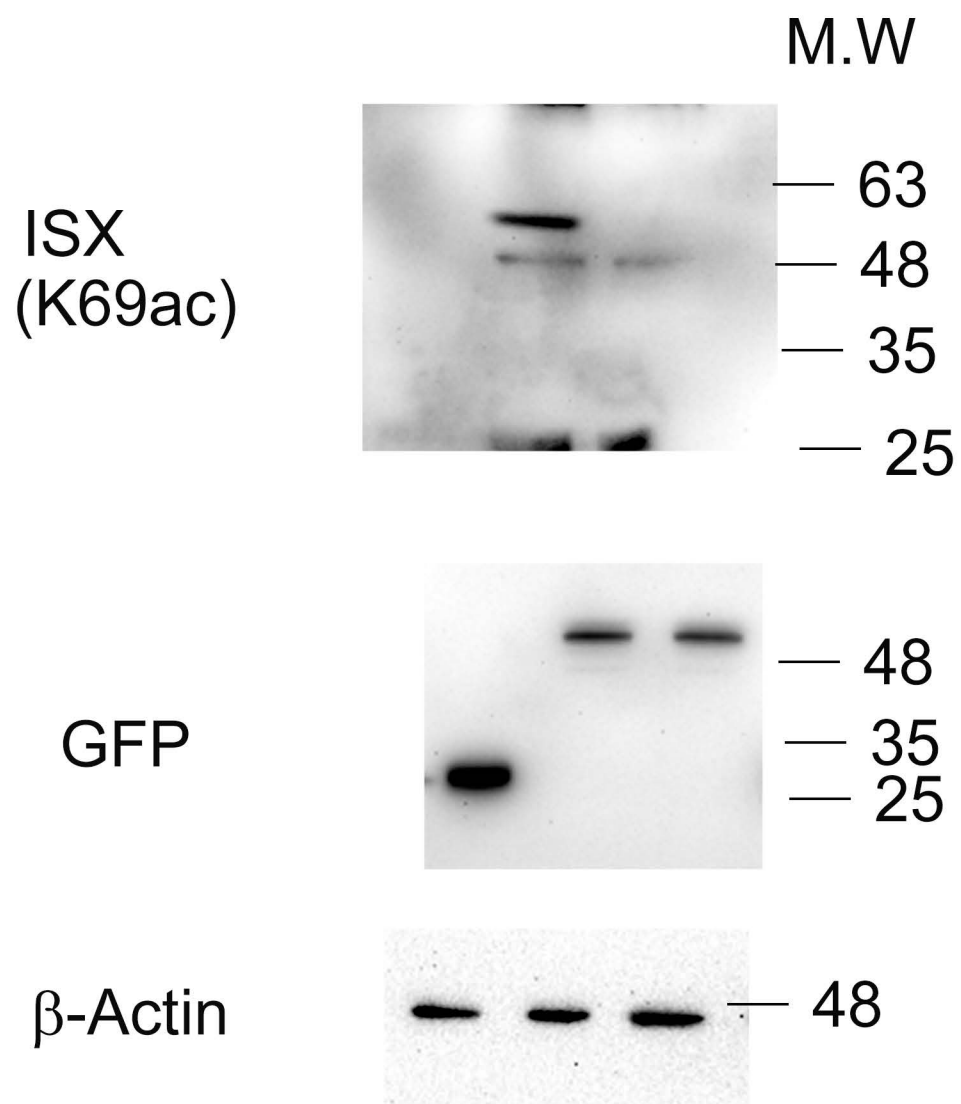

Figure EV3e

Supplement: Supplementary file 2 — Source Data for Expanded View [file EMBR-21-e48795-s009.zip › Source_Data_for_EV_Figures/Source_Data_Figure_EV3.pdf]

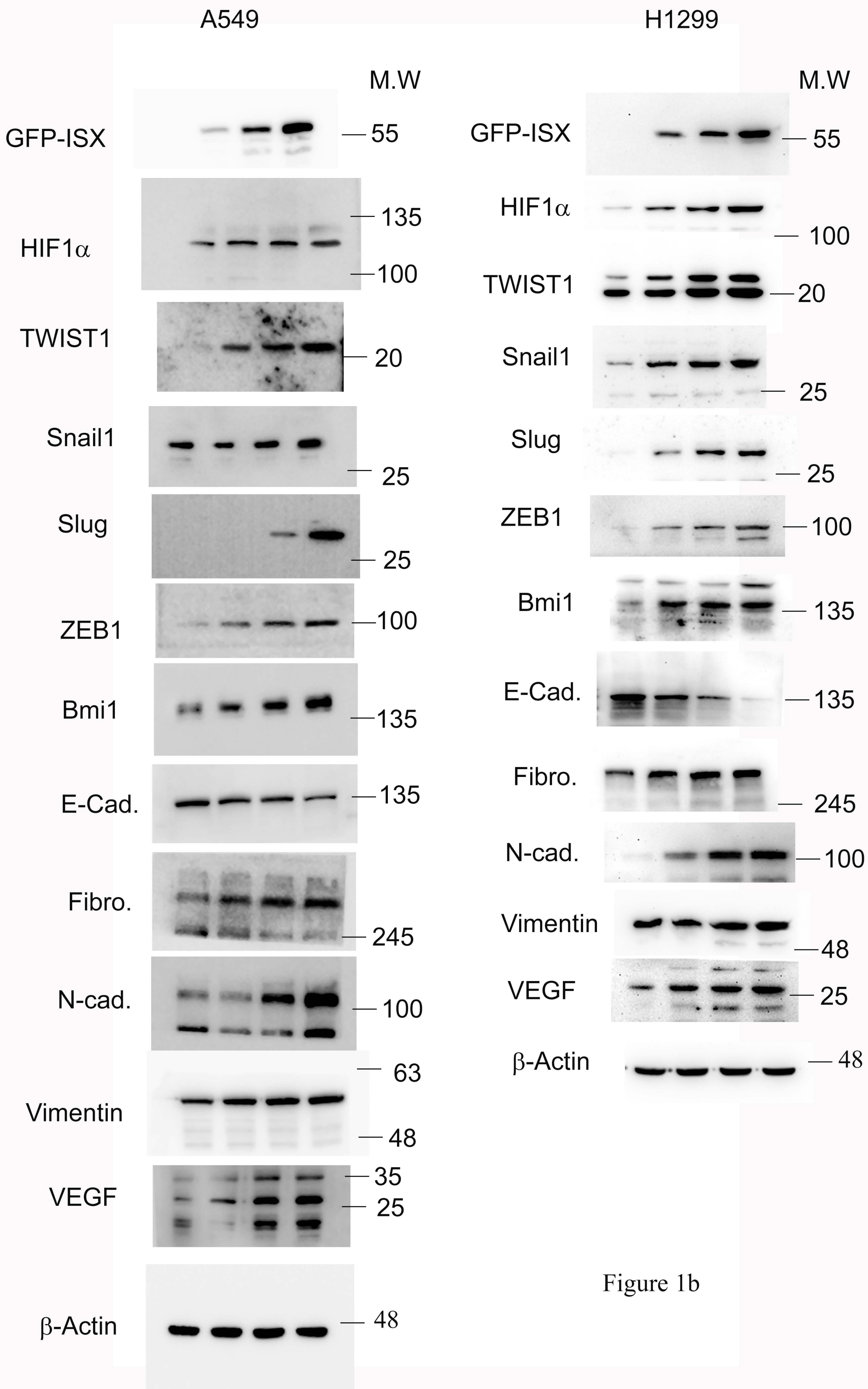

Supplement: Supplementary file 4 — Source Data for Figure 1 [file EMBR-21-e48795-s002.pdf]

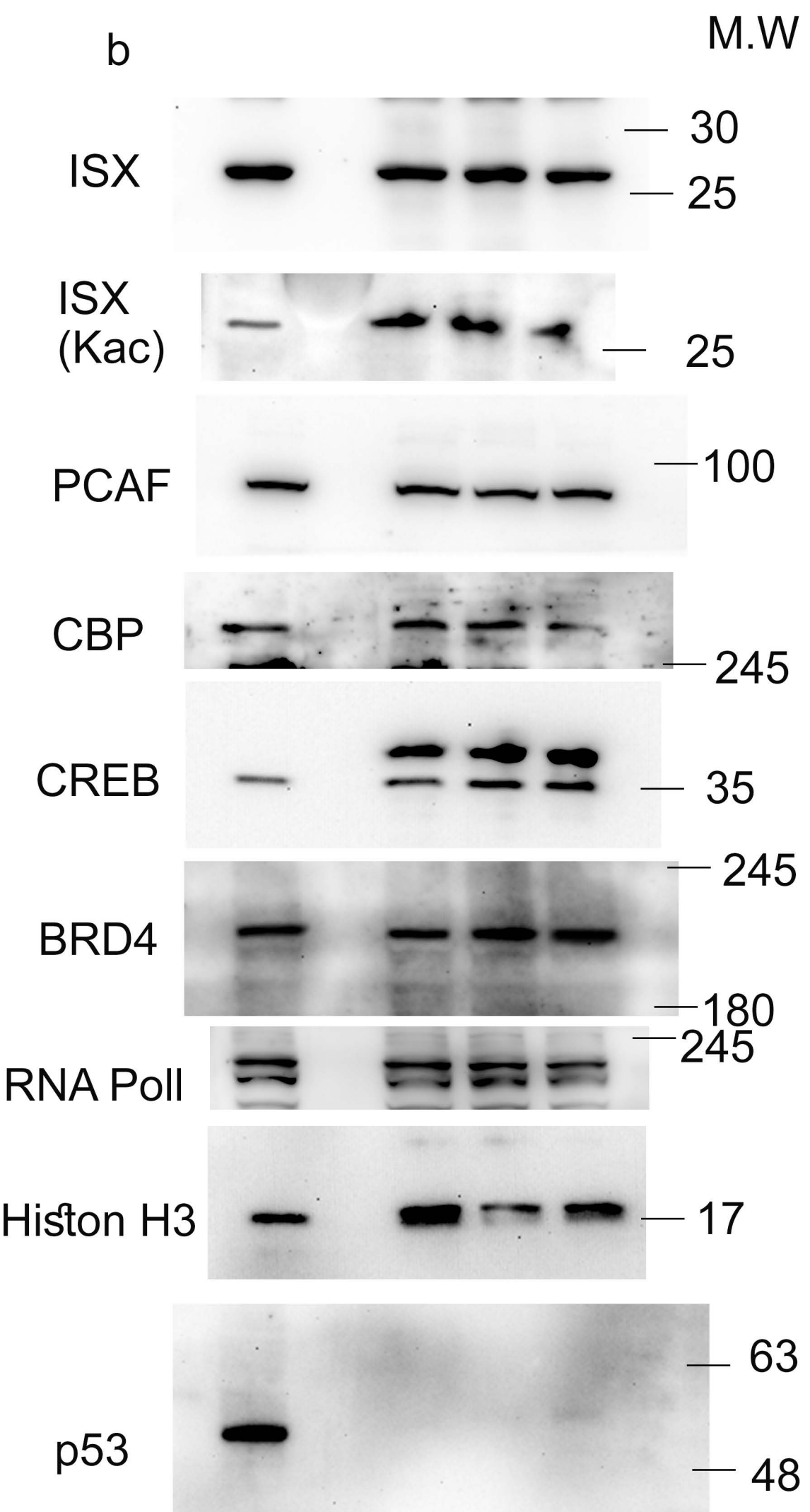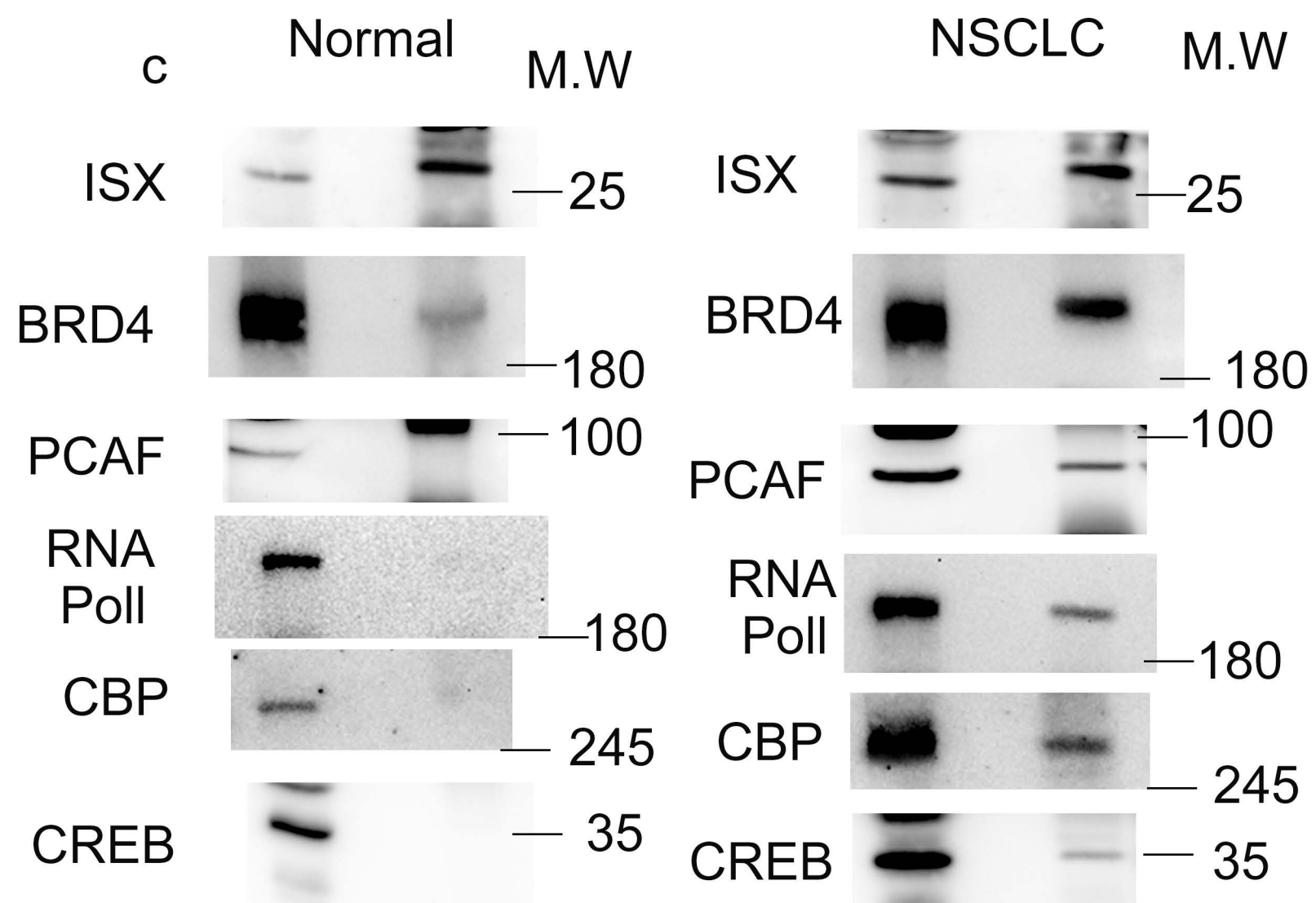

Figure 2b and c

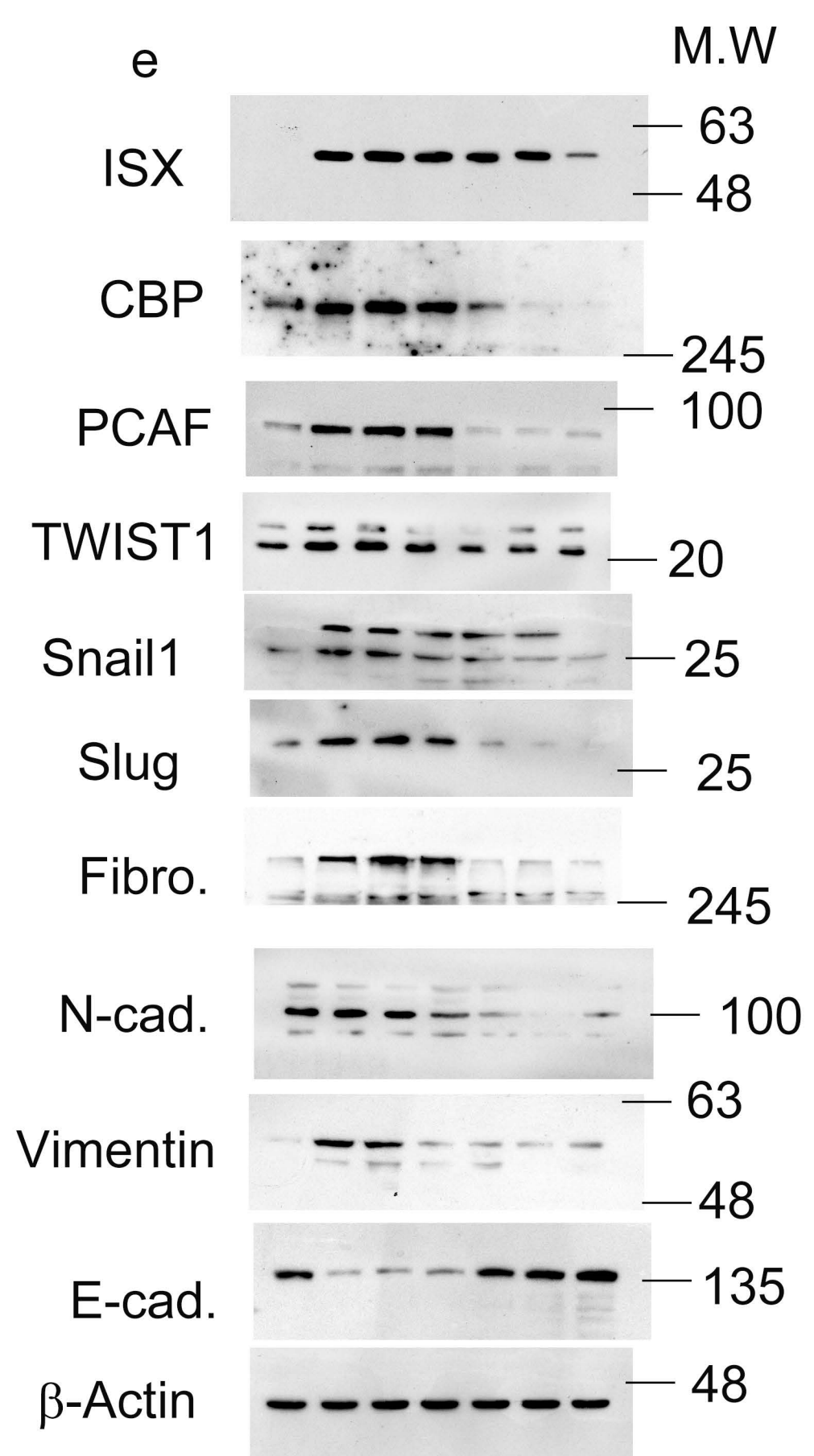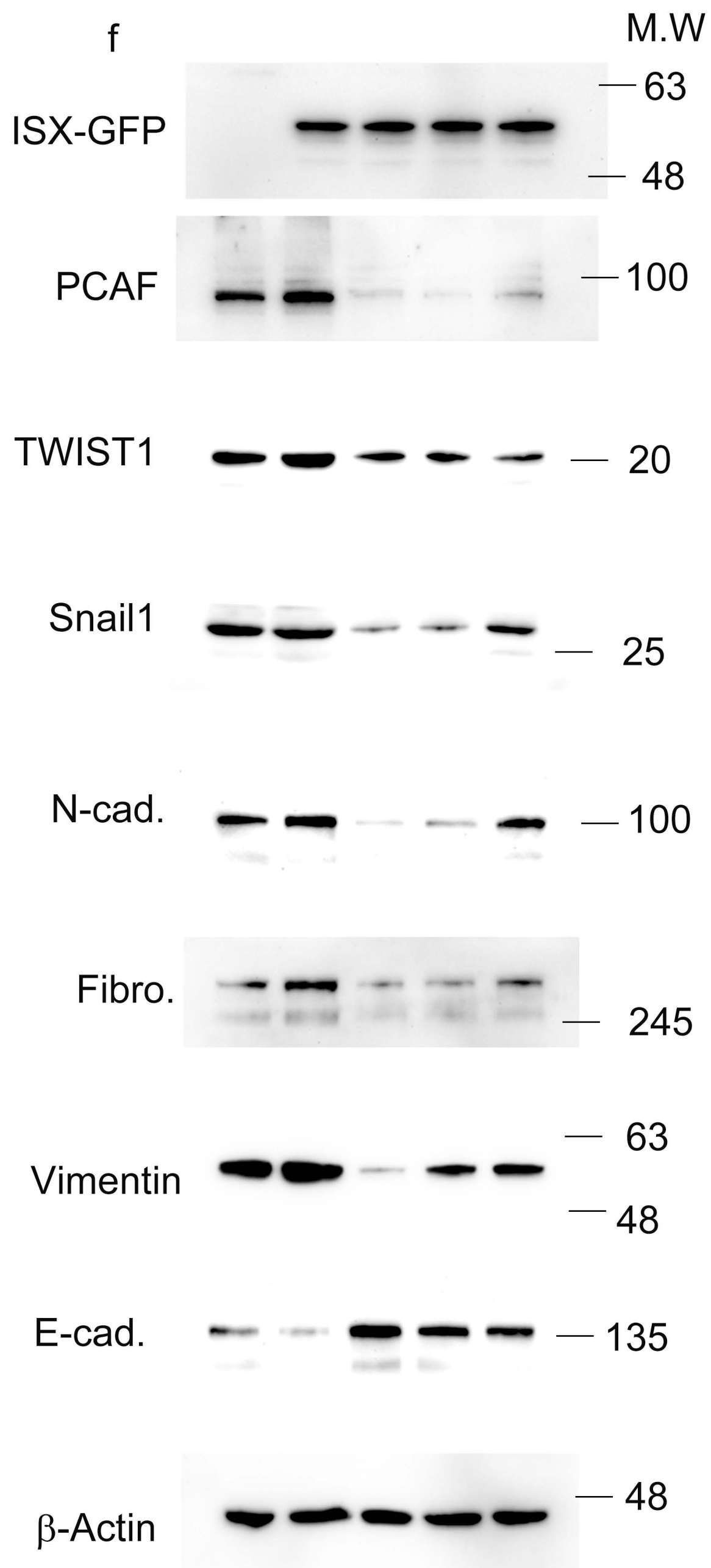

Figure 2e and f

Supplement: Supplementary file 5 — Source Data for Figure 2 [file EMBR-21-e48795-s003.pdf]

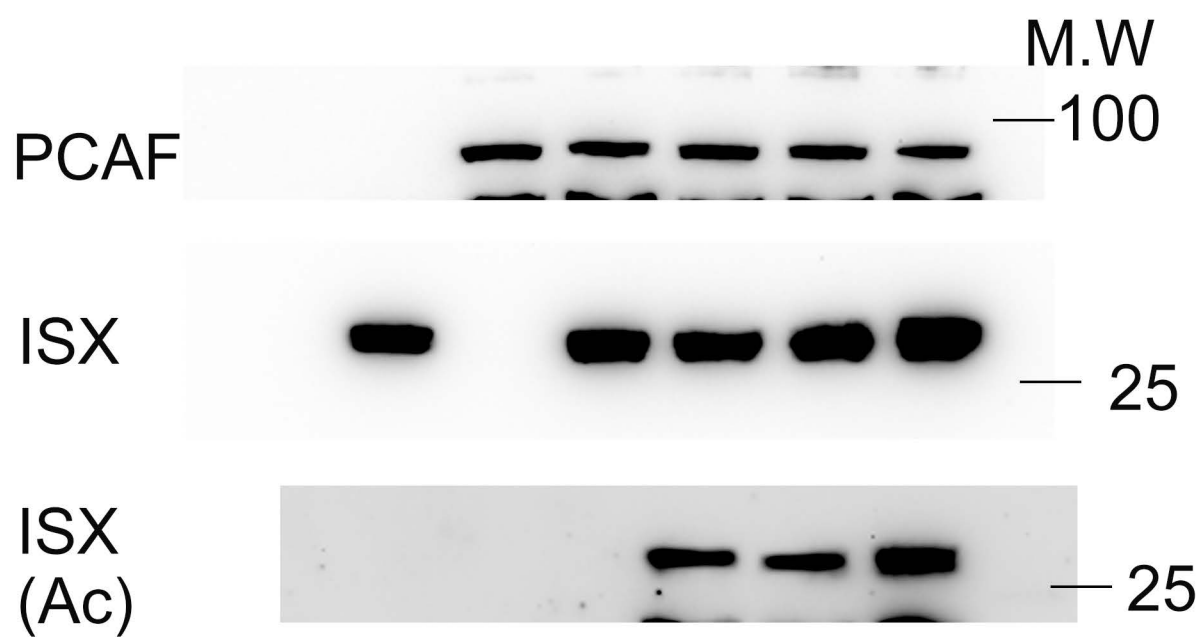

Figure 3c

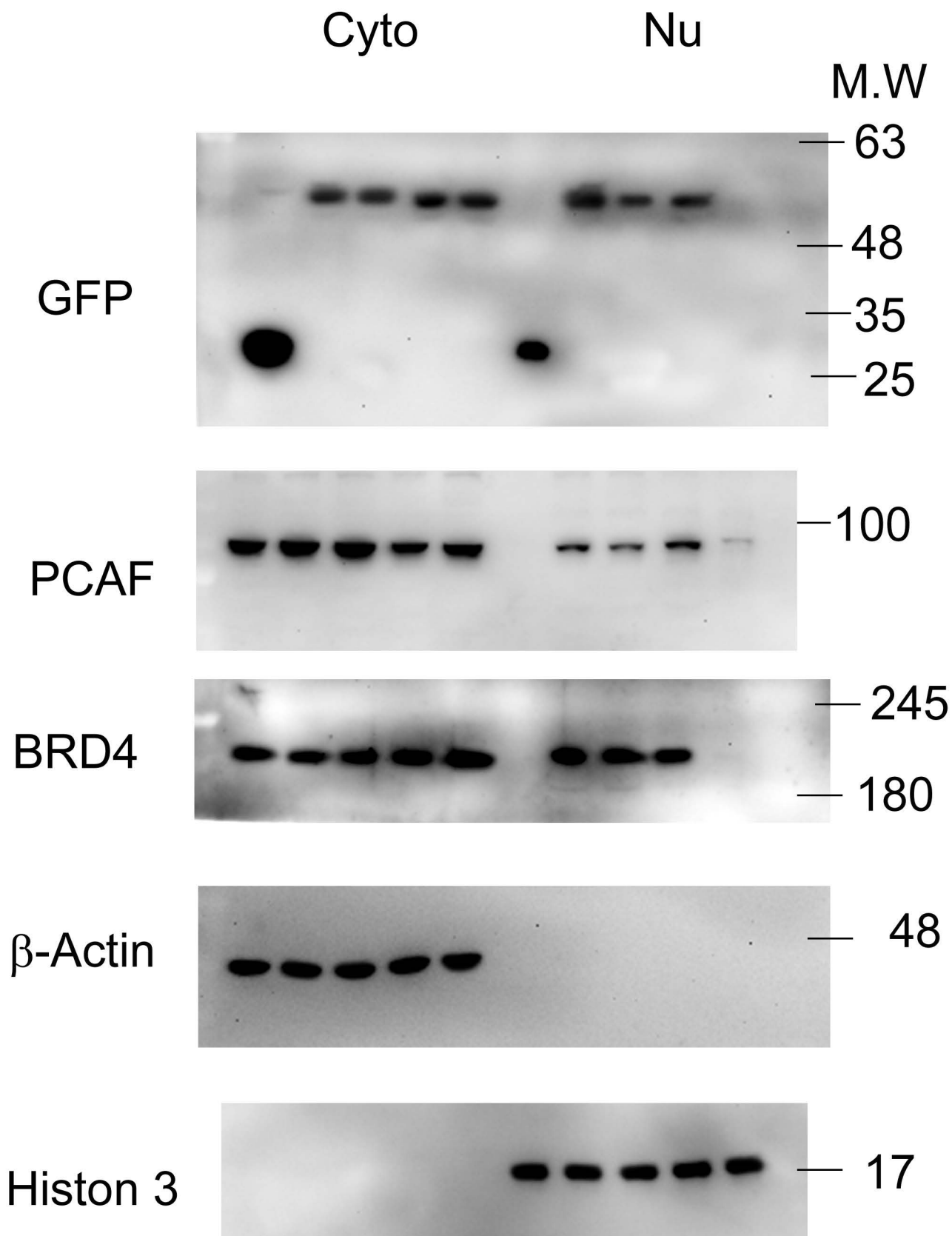

Figure 3d

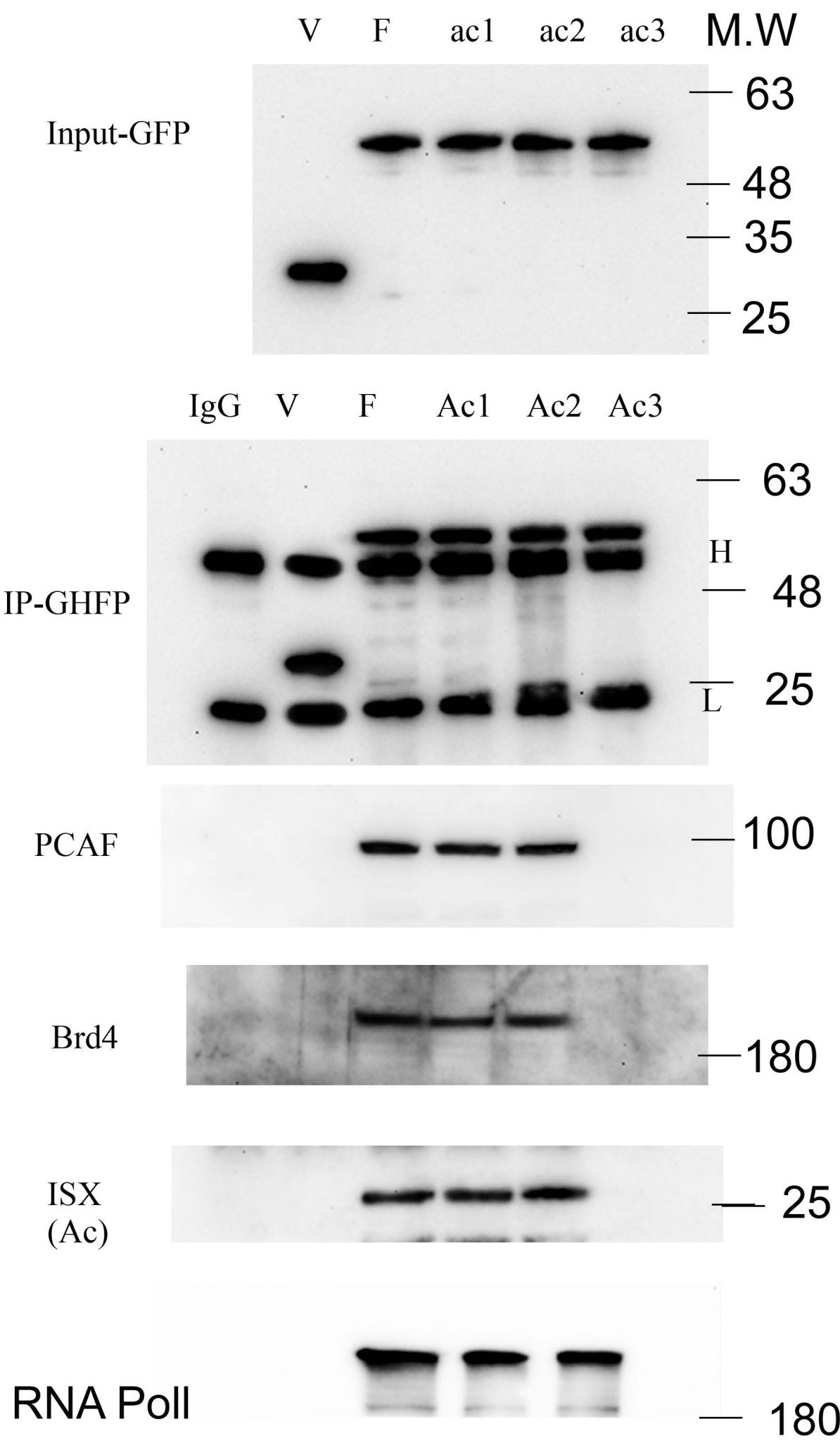

Figure 3e

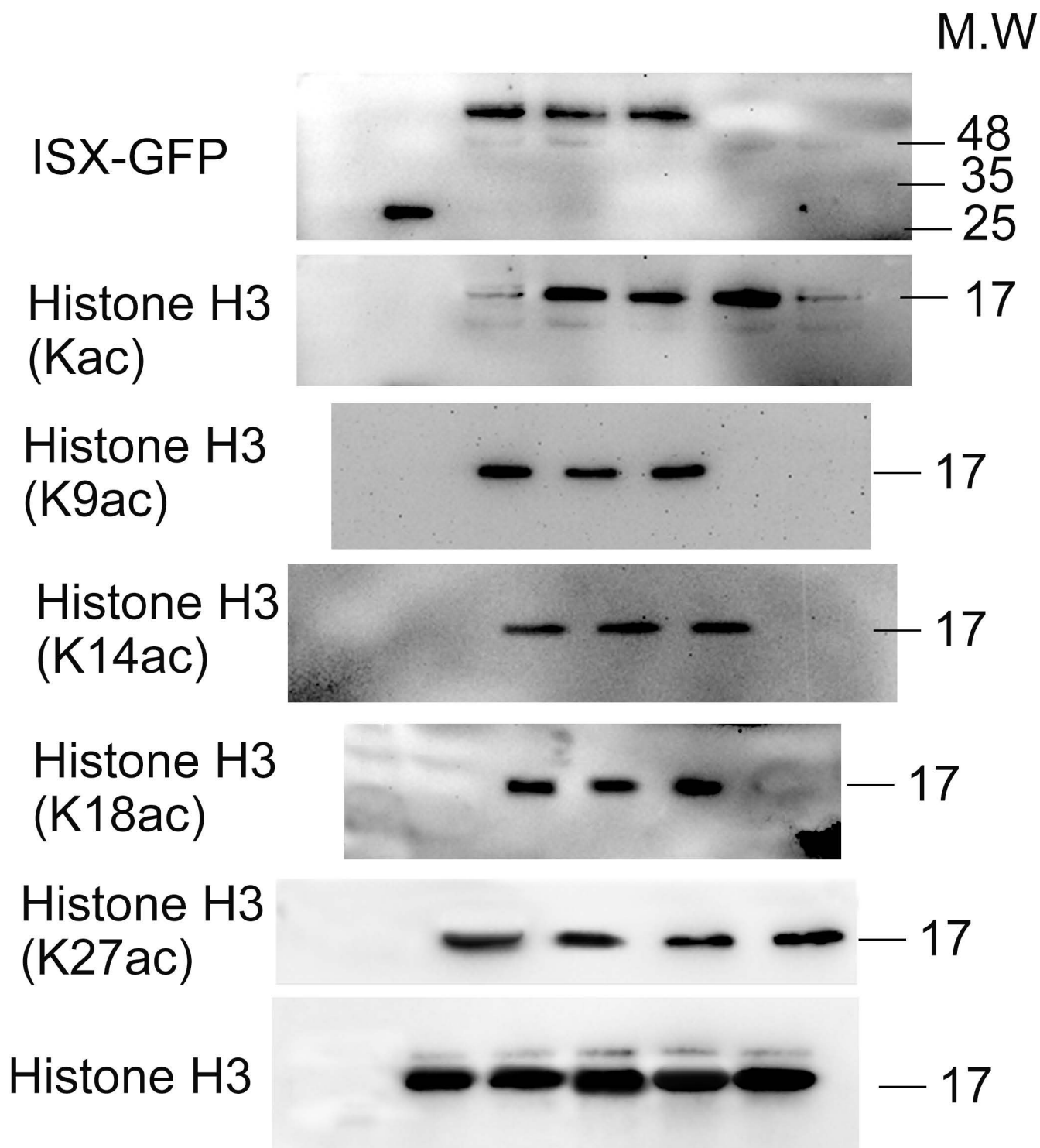

Figure 3f

Supplement: Supplementary file 6 — Source Data for Figure 3 [file EMBR-21-e48795-s004.pdf]

Figure 4c and d

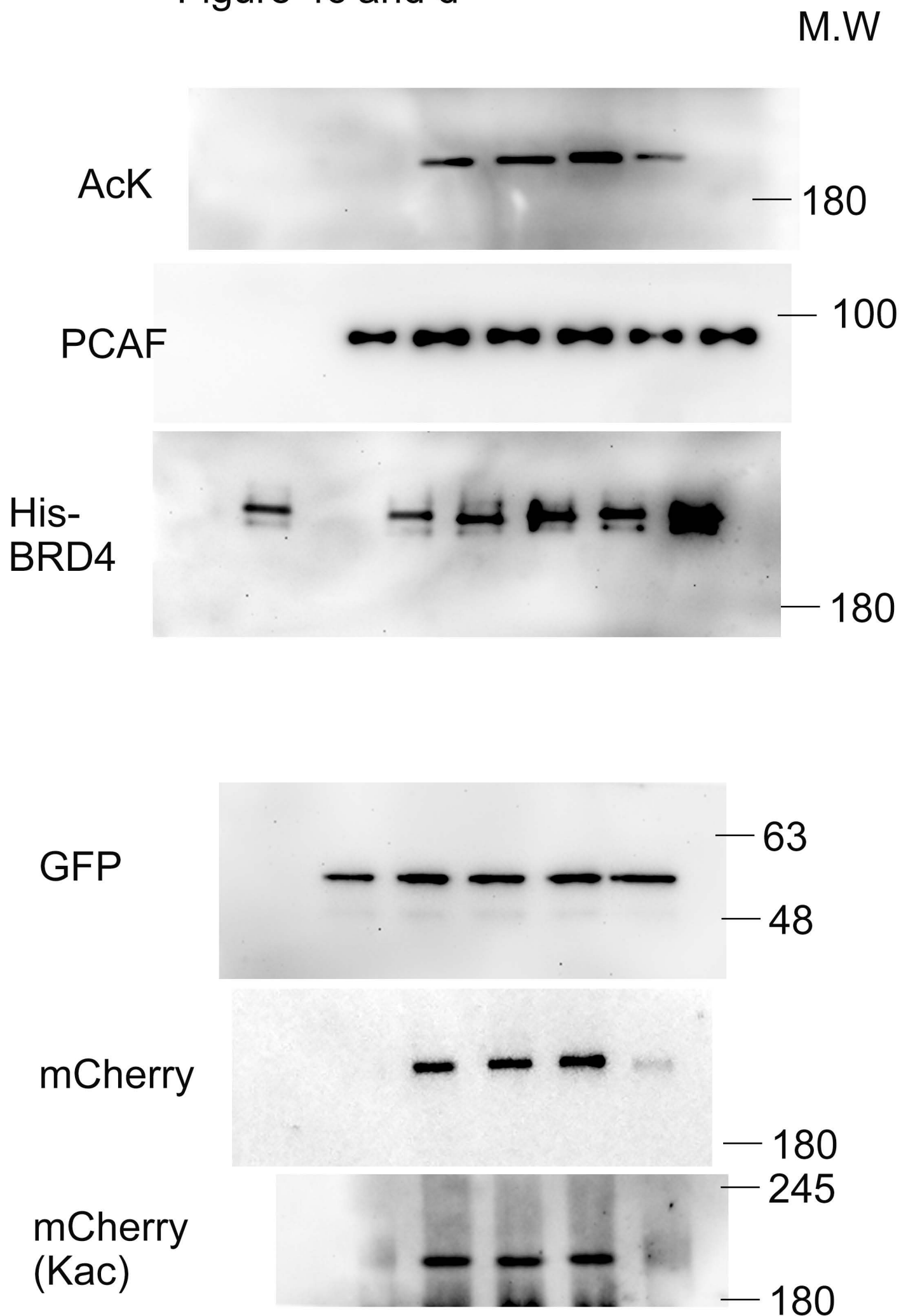

Supplement: Supplementary file 7 — Source Data for Figure 4 [file EMBR-21-e48795-s005.pdf]

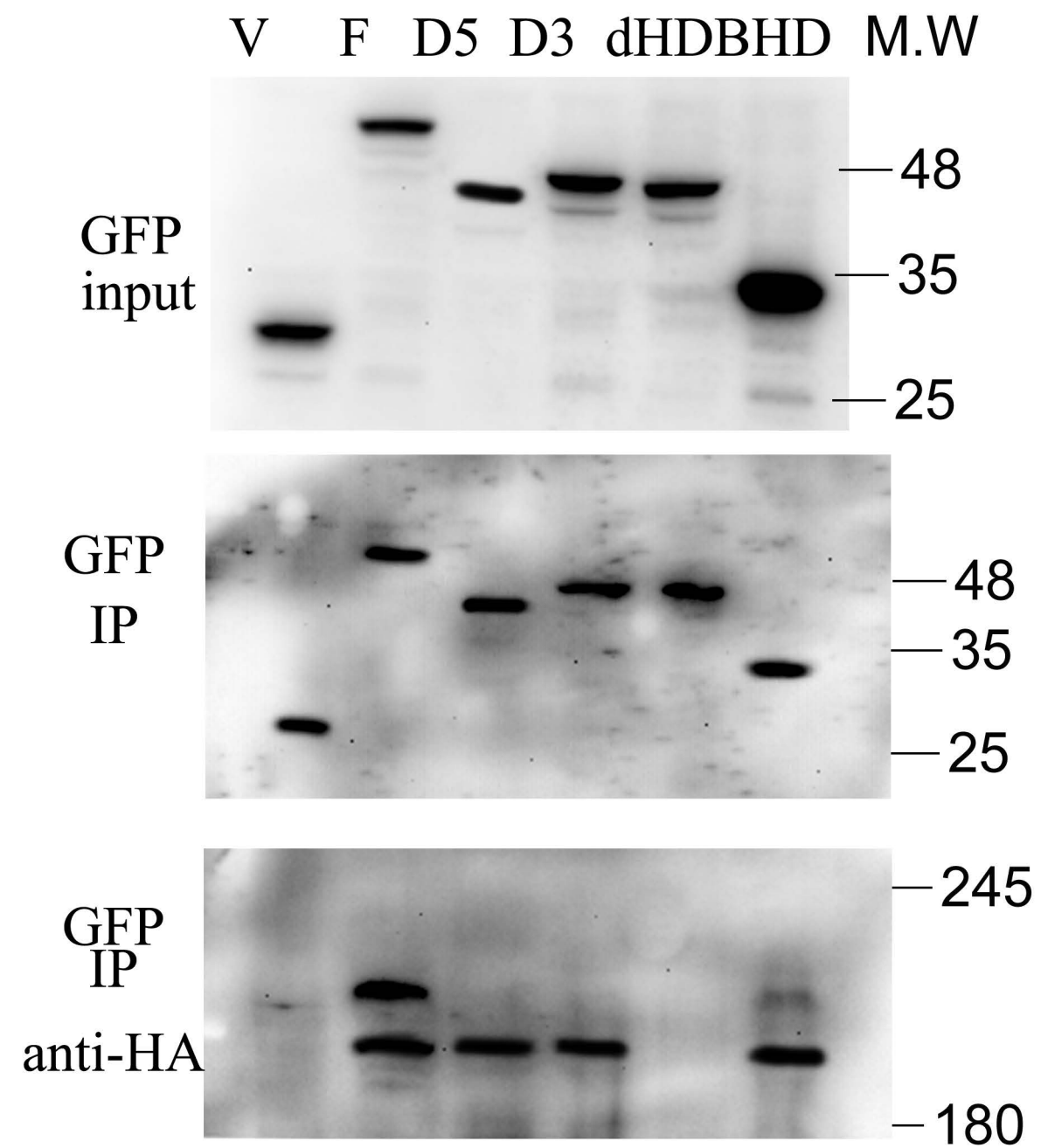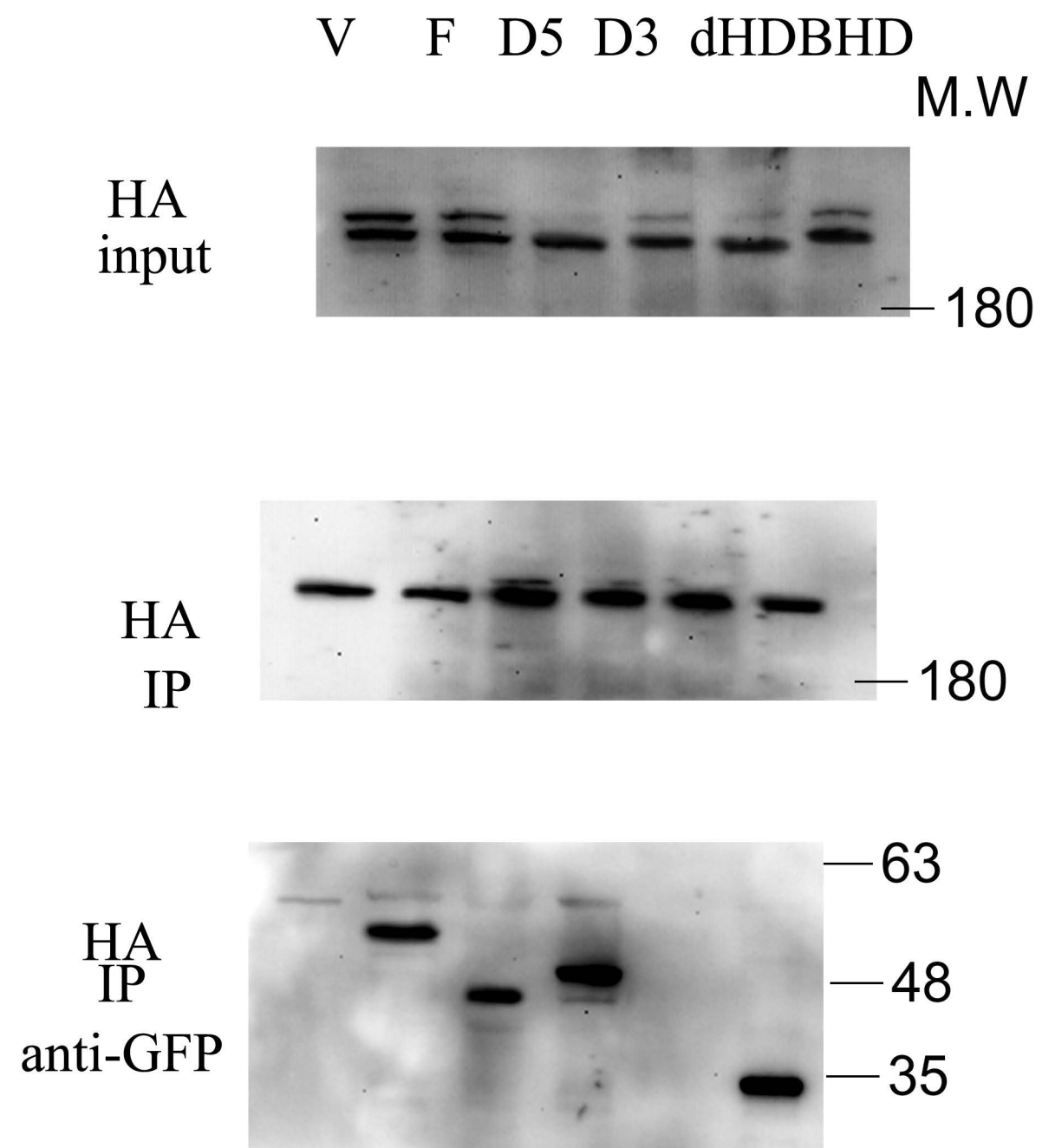

Figure 5a and b

Supplement: Supplementary file 8 — Source Data for Figure 5 [file EMBR-21-e48795-s006.pdf]

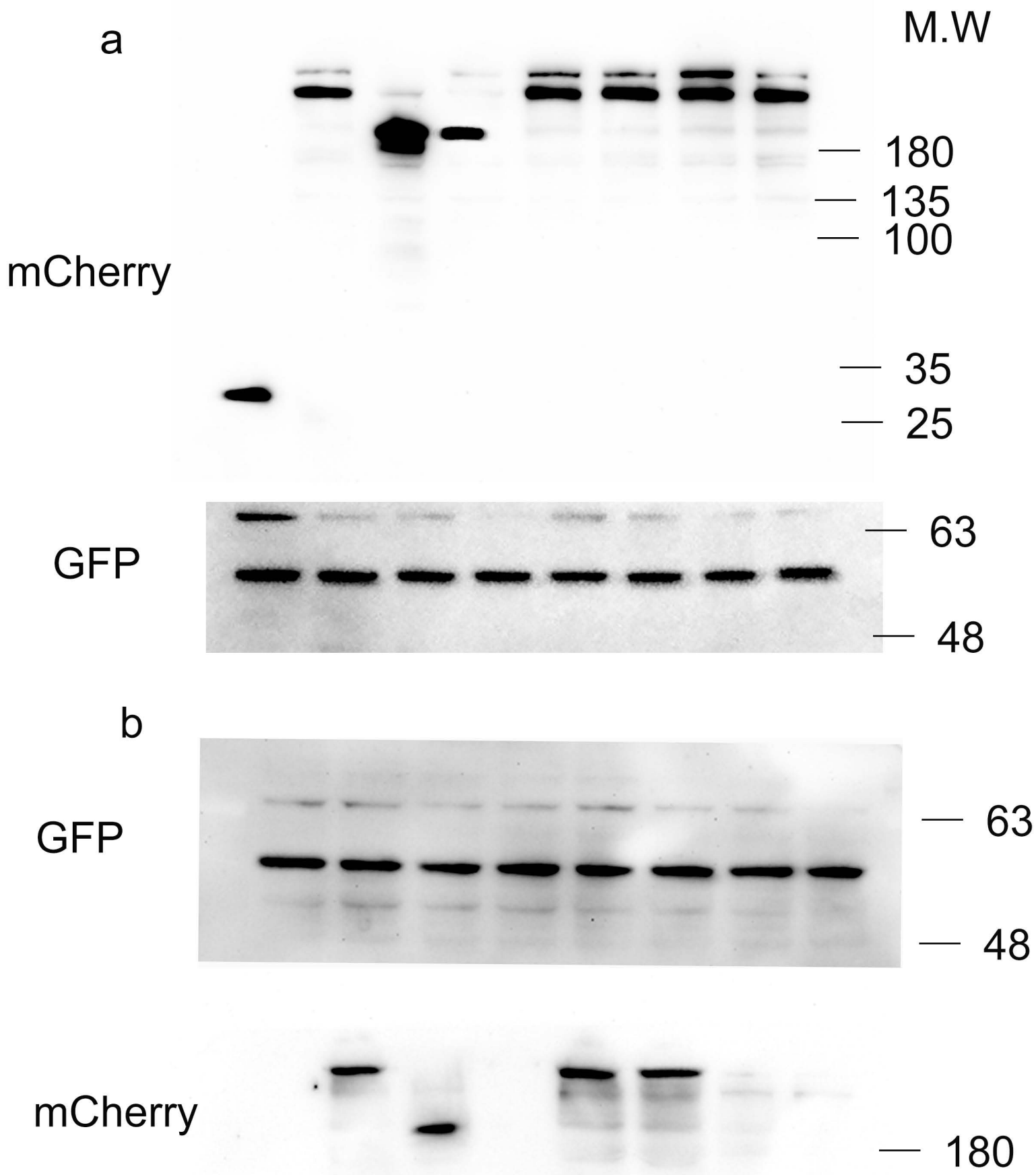

Figure 6a and b

Supplement: Supplementary file 9 — Source Data for Figure 6 [file EMBR-21-e48795-s007.pdf]

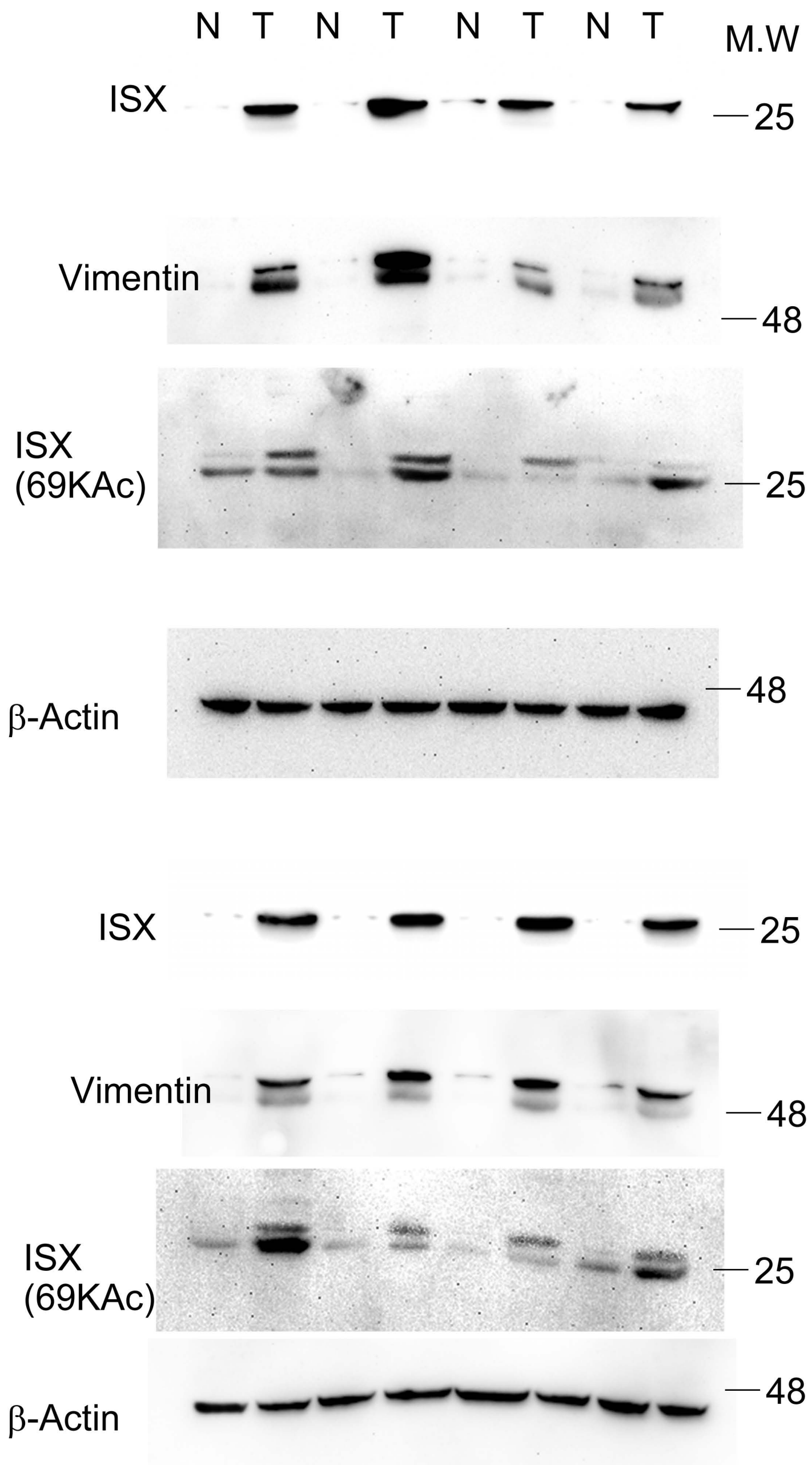

Supplement: Supplementary file 10 — Source Data for Figure 7 [file EMBR-21-e48795-s008.pdf]
